# Intravenous transplantation of amnion-derived mesenchymal stem cells promotes functional recovery and alleviates intestinal dysfunction after spinal cord injury

**DOI:** 10.1371/journal.pone.0270606

**Published:** 2022-07-08

**Authors:** Soichiro Takamiya, Masahito Kawabori, Kazuyoshi Yamazaki, Sho Yamaguchi, Aki Tanimori, Koji Yamamoto, Shunsuke Ohnishi, Toshitaka Seki, Kotaro Konno, Khin Khin Tha, Daigo Hashimoto, Masahiko Watanabe, Kiyohiro Houkin, Miki Fujimura

**Affiliations:** 1 Department of Neurosurgery, Graduate School of Medicine, Hokkaido University, Sapporo, Hokkaido, Japan; 2 Regenerative Medicine and Cell Therapy Laboratories, Kaneka Corporation, Kobe, Hyogo, Japan; 3 Department of Gastroenterology and Hepatology, Graduate School of Medicine, Hokkaido University, Sapporo, Hokkaido, Japan; 4 Laboratory of Molecular and Cellular Medicine, Faculty of Pharmaceutical Sciences, Hokkaido University, Sapporo, Hokkaido, Japan; 5 Department of Anatomy and Embryology, Graduate School of Medicine, Hokkaido University, Sapporo, Hokkaido, Japan; 6 Global Center for Biomedical Science and Engineering, Hokkaido University Faculty of Medicine, Sapporo, Hokkaido, Japan; 7 Department of Hematology, Graduate School of Medicine, Hokkaido University, Sapporo, Hokkaido, Japan; Government College University Faisalabad, PAKISTAN

## Abstract

Spinal cord injury (SCI) is often accompanied by gastrointestinal dysfunction due to the disconnection of the spinal autonomic nervous system. Gastrointestinal dysfunction reportedly upregulates intestinal permeability, leading to bacterial translocation of the gut microbiome to the systemic circulation, which further activates systemic inflammation, exacerbating neuronal damage. Mesenchymal stem cells (MSC) reportedly ameliorate SCI. Here, we aimed to investigate their effect on the associated gastrointestinal dysfunction. Human amnion-derived MSC (AMSCs) were intravenously transplanted one day after a rat model of midthoracic SCI. Biodistribution of transplanted cells, behavioral assessment, and histological evaluations of the spinal cord and intestine were conducted to elucidate the therapeutic effect of AMSCs. Bacterial translocation of the gut microbiome was examined by *in situ* hybridization and bacterial culture of the liver. Systemic inflammations were examined by blood cytokines, infiltrating immune cells in the spinal cord, and the size of the peripheral immune tissue. AMSCs released various neurotrophic factors and were mainly distributed in the liver and lung after transplantation. AMSC-transplanted animals showed smaller spinal damage and better neurological recovery with preserved neuronal tract. AMSCs transplantation ameliorated intestinal dysfunction both morphologically and functionally, which prevented translocation of the gut microbiome to the systemic circulation. Systemic inflammations were decreased in animals receiving AMSCs in the chronic phase. Intravenous AMSC administration during the acute phase of SCI rescues both spinal damage and intestinal dysfunction. Reducing bacterial translocation may contribute to decreasing systemic inflammation.

## Introduction

Spinal cord injury (SCI) is an unresolved public health issue with an annual incidence of 40–80 per million people [1]. In addition to the paralysis caused by SCI, bowel dysfunction and systemic infections are common symptoms after SCI, especially in the acute phase. Recent studies revealed that weakening of peristalsis due to the damaged spinal autonomic nervous system [2–6] or exaggerated reflex activity of sympathetic preganglionic neurons [7] might provoke bowel dysfunction after SCI. These neurological dysfunctions subsequently impair the intestinal barrier function and aggravate intestinal permeability, leading to the translocation of gut microbiome bacteria into the systemic circulation. Activated systemic inflammation further exacerbates neuronal damage. Dysbiosis of the intestinal tract also adversely influences the recovery of SCI in the chronic phase through immune suppression [8–10].

Stem cell therapy is a promising treatment for SCI [11, 12]. Stem cells are involved in various mechanisms to ameliorate neurological deficits [11]; however, data regarding the role of stem cell therapy in bowel dysfunction after SCI is scarce, and the underlying mechanisms are unknown. This study aimed to determine whether administration of amnion-derived mesenchymal stem cells (AMSCs) alleviates gastrointestinal dysfunction after SCI and prevents gut bacterial translocation into the systemic circulation, ameliorating systemic inflammation and neurological sequelae.

## Methods

A full description of the Methods can be found in the S1 File digital content. Animal protocols were approved by the Animal Studies Ethics Committee of the Hokkaido University Graduate School of Medicine (approval number: 17–0065). All experimental procedures were conducted following the Institutional Guidelines for Animal Experimentation and the Guidelines for Proper Conduct of Animal Experiments by the Science Council of Japan, and every effort was made to minimize pain and discomfort to the animals.

### AMSC preparation and measurement of trophic factors

Frozen human AMSC vials were provided by Kaneka Corporation (Osaka, Japan). After thawing, 9.5×10^4^/mL of AMSC suspension or medium without AMSCs were cultured in the collagen-coated 6-well plates, and the cell culture supernatants or medium were collected 24 h later (n = 3). Brain-derived neurotrophic factor (BDNF), vascular endothelial growth factor (VEGF), basic fibroblast growth factor (b-FGF), hepatocyte growth factor (HGF), and beta-neuro growth factor (β-NGF) were measured for evaluating the secretion potential of neurotrophic factors [11], and R-Spondin 1 were evaluated for intestinal trophic factor using ELISA kits [13].

### Animals, SCI model, and cell transplantation

Thoracic (T6-7) SCI models were created with 9-week-old female Sprague-Dawley rats (CLEA Japan, Inc., Japan) by pinching the spinal cord extradurally using a modified aneurysm clip (MIZUHO, Japan) for 1 min [14]. General anesthesia was induced using 5% isoflurane in 70% N_2_O and 30% O_2_ gas, followed by the maintenance of anesthesia with 1.5–2% isoflurane in 70% N_2_O and 30% O_2_ gas to establish an SCI model. Animals were randomly separated into two groups 24 h after SCI; AMSC (1×10^7^ AMSCs in 1 mL PBS) or PBS group (1 mL PBS), and were intravenously transplanted through the tail vein with the injection rate of 0.5 mL/min. The animals were sacrificed at various time points via an isoflurane overdose as described previously [15, 16].

The transplanted cells’ distribution was investigated using a chemiluminescence imaging system (FUSION FX7 EDGE, Vilber Lourmat, France) [17, 18]. Imaging was carried out 2 h post-transplantation and daily thereafter for 7 d. For the *ex vivo* imaging, data of the spinal cord, intestinal tract, thymus, lungs, liver, spleen, and kidneys were acquired 2 h post-transplantation and analyzed using the Evolution Capture EDGE software (Vilber, France). Cell distributions were further confirmed by immunohistochemistry by investigating positive cells of anti-Ku80 antibody (1:500, ab80592; Abcam, Cambridge, UK) in the spinal cord, lungs, liver, and ileum.

### Neurological, histopathological, and radiographical evaluation of spinal cord after cell transplantation

Basso-Beattie-Bresnahan (BBB) scale was assessed weekly until 28 d after SCI [14, 19] for neurological evaluation. Kluver-Barrera staining referring to the absence of Luxol fast blue (LFB) was performed 28 d after SCI to evaluate the length of the injured spinal cord [14, 20]. For the measurement of trophic factors, the spinal cords were collected 3 d after SCI and were cut into 10-mm length sections with a central focus on the injured lesions. Subsequently, the proteins were extracted to quantitively measure BDNF, VEGF, and b-FGF using commercially available ELISA kits (total BDNF, human VEGF, and human bFGF; R&D Systems, Inc., MN, USA) according to the manufacturer’s protocols. Spinal cord MRI was conducted before SCI induction and weekly thereafter until 21 d after SCI to assess neuronal connectivity. Fractional anisotropy (FA) value, axial diffusivity (AD), and radial diffusivity (RD) maps were calculated from diffusion tensor imaging (DTI) obtained by a 3.0 T magnetic resonance (MR) scanner (Magnetom Prisma, Siemens Health Care, Germany) [21], where FA refers to the anisotropy of diffusion, AD represents the directional diffusivity along the axonal pathway, and RD represents the diffusivity along the orthogonalized axonal pathway [22].

### Histological analysis of gut after SCI with/without cell transplantation

Hematoxylin and eosin (H&E) and periodic acid-Schiff (PAS) staining of the ileum was performed 3 and 14 d after SCI [23]. The villus height, villus density (the number of villi per millimeter), crypt depth, and muscle layer thickness were measured by H&E staining for assessing morphological change after SCI with/without cell transplantation, and PAS staining was performed to assess their abilities of the goblet cells to produce mucus as for functional analysis. The permeability of the ileum was assessed 3 d after SCI using anti-zonula occludens 1 (zo-1) antibody (1:1000, 61–7300; Invitrogen Life Technologies, Germany) to detect the integrity of tight junction protein [24]. Intestinal peristalsis was evaluated 3 and 14 d after SCI by assessing intestinal neurotransmitters; nNOS (1:400, 4231; Cell Signaling Technology Inc., MA, USA) [25]. Measurements were performed by automated cell/area counter (BZ-X Analyzer, Keyence Co., Osaka, Japan) under a magnification of 100× in five non-overlapping fields.

### Bacterial translocation analysis and evaluating systemic inflammation

To evaluate whether cell transplantation ameliorates translocation of the intestinal tract bacteria after SCI, fluorescence *in situ* hybridization (FISH) for the bacterial mRNA in the submucosal layer of ileum 3 and 7 d after SCI [26], and liver culture 7 d after SCI were performed [9]. Microbiota sequencing was further assessed to evaluate the change of gut microbiota by collecting feces and small intestinal contents 3 and 14 d after SCI. 16S rRNA gene expressions were amplified with MiSeq Reagent Kit V3 (Illumina, CA, USA) and evaluated with microbial diversity analysis using the Qiime followed by Simpson’s index.

Inflammation cytokines, IL-6 and IFN-γ, were quantified from blood samples of the animals up to 7 d after SCI using commercial ELISA kits for detecting acute systemic inflammation. Chronic systemic inflammations were evaluated by the change of the thymus size [14], and by immunohistochemistry of macrophages in the spinal cord (anti-CD68 antibody, 1:500, MCA341GA; Bio-Rad Laboratories, Inc., CA, USA) [27, 28] at 28 d after SCI

### Statistical analyses

All assessments were performed by blinded investigators. Data are expressed as the mean ± standard error of the mean. Statistical analyses were performed using JMP Pro 14 software (SAS Institute Inc., NC, USA). The sample size was chosen based on comparable experiments from our previous experiments [14]. Statistical comparisons between two different groups were made using the Wilcoxon rank-sum test except for the trophic factor assessment, performed using the Shapiro-Wilk test, followed by Welch’s *t*-test. Probability values of *P*<0.05 were considered statistically significant.

## Results

### AMSCs release various neurotrophic factors

**Fig 1:**
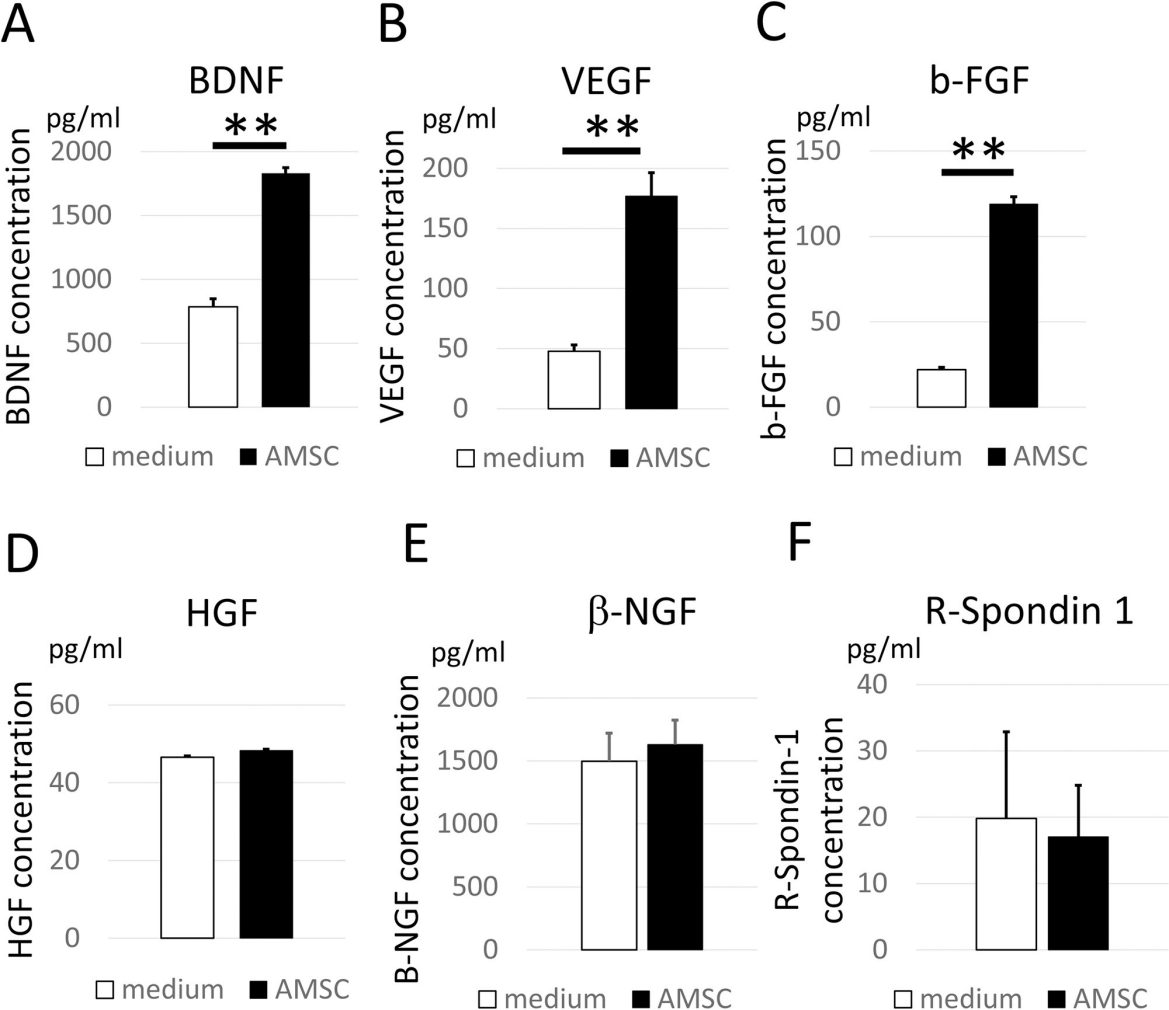
AMSCs secreted neurotrophic factors *in vitro*. The ability of AMSCs to secrete trophic factors was evaluated using ELISA. Neurotrophic factors such as (A) BDNF, (B) VEGF, and (C) b-FGF were significantly secreted from AMSCs compared with the control medium. Other neurotrophic factors such as (D) HGF and (E) β-NGF were secreted from AMSCs in negligible amounts. Intestinal trophic factors (F) R-Spondin 1 were also barely secreted. Data are presented as the mean ± standard error (SE). **, *P*<0.01. AMSC, amnion-derived mesenchymal stem cell; BDNF, brain-derived neurotrophic factor; b-FGF, basic fibroblast growth factor; HGF, hepatocyte growth factor; NGF, nerve growth factor; VEGF vascular endothelial growth factor.

Neuronal and intestinal trophic factors released in the culture medium were examined to evaluate the therapeutic potential of AMSC. AMSCs secreted significantly higher amount of BDNF, VEGF, and b-FGF; however, there was no differences in HGF and β-NGF, nor intestinal trophic factor, R-Spondin-1 (Fig 1A–1F). These results indicate that the AMSCs possess a high potential for releasing trophic factors necessary for neuroprotection.

### Intravenously administered AMSC mainly distributes in the liver and lungs

**Fig 2:**
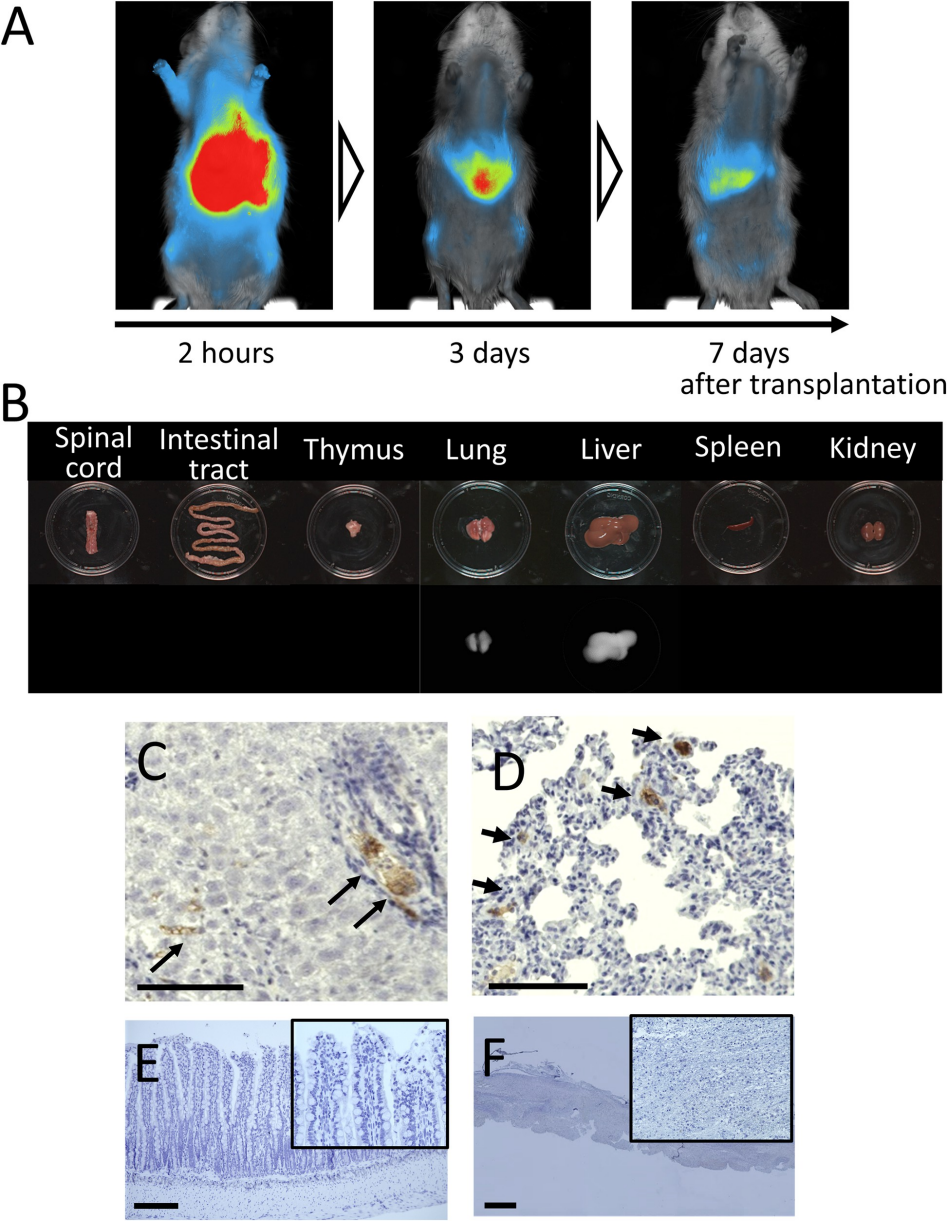
Transplanted AMSCs were mainly distributed in the liver and lung. (A) *In vivo* imaging was conducted to evaluate the distribution of the infused AMSCs. Fluorescence from the XenoLight DiR labeled AMSCs was detected 2 h, 3 d, and 7 d after transplantation. Most of the signals were found in the upper abdomen. (B) *Ex vivo* imaging 7 d after transplantation (left: Spinal cord, intestinal tract, thymus, lung, liver, spleen, and kidney). The bright-field images (Upper row) and fluorescent images (Lower row) were shown. Fluorescence was detected in the lungs and liver, but it was not detected in the spinal cord, intestinal tract, thymus, spleen, and kidneys. (C–F) Histopathological findings 7 d after transplantation. To validate the findings in the *in vivo* and *ex vivo* assays, the lungs and liver were histopathologically assessed for the existence of the AMSCs. The Ku80-positive cells were detected in the liver (C) and the lungs (D) of the AMSC group (magnification, ×200; scale bar: 100 μm). On the other hand, Ku80-positive cells were not detected in the ileum from AMSC group 3 d following SCI (E; magnification, ×100 and 200; scale bar: 200 μm), nor in the spinal cord 2 h following SCI (F; magnification, ×20 and 200; scale bar: 1 mm).

Immediately after transplantation, AMSCs were strongly detected around the upper abdomen and remained detectable for 1 week (Fig 2A). *Ex vivo* imaging revealed that cells were primarily distributed in the liver and lungs but absent in the spinal cord and intestinal tract (Fig 2B). These findings were further confirmed by immunostaining. Transplanted cells were detected in the lungs and liver in the AMSC group up to 7 d post-transplantation (Fig 2C and 2D). No cell was found in the ileum and spinal cord (Fig 2E and 2F). The results show that AMSCs do not have to accumulate in the injured area to promote functional recovery.

### AMSCs transplantation improves neurological function after SCI

**Fig 3:**
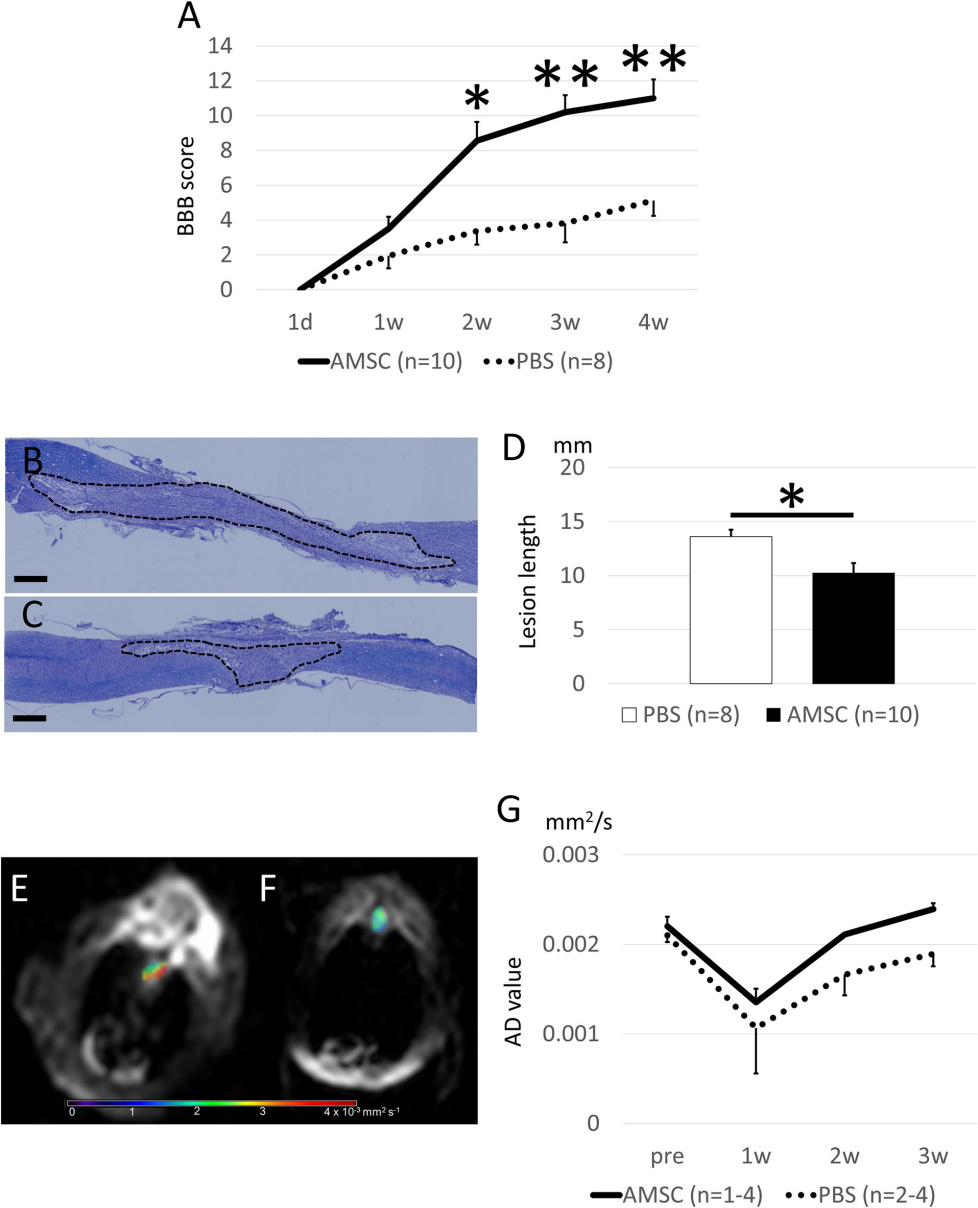
Administration of AMSCs ameliorated the degree of spinal cord damage. (A) Locomotor function was evaluated the next day and weekly thereafter until 4 weeks after the SCI by BBB score. The scores were significantly higher on and after the second week after SCI in the AMSC group compared with the PBS group (*P* = 0.0267, 0.0089, and 0.0051, respectively). (B–D) The Kluver-Barrera staining of the spinal cord 4 weeks after SCI showed the degree of spinal cord damage (the dotted area). AMSC Groups (B) and PBS group (C) were evaluated (scale bar: 1 mm). The mean lesion lengths in the AMSC group were significantly shorter than that in the PBS group (*P* = 0.0386). (E–G) The spinal cord AD value of the injured lesion was evaluated via diffusion tensor imaging. The representative images of the AMSC group (E) and PBS group (F) are shown. The AD value was temporarily decreased a week after SCI, which then gradually recovered in both groups. The AD values in the AMSC group were higher than those in the PBS group (*P* = 0.0638). Data are presented as mean ± standard error (SE). *, *P*<0.05 vs PBS group; **, *P*<0.01 vs PBS group.

AMSC group presented a significantly better BBB score from 2 weeks post-transplantation than the PBS group (Fig 3A), and lesions in the AMSC group were significantly shorter than those in the PBS group 4 weeks post-SCI (Fig 3B–3D). The level of BDNF in the spinal cords of the AMSC group was slightly higher than in the PBS group, though it was not statistically significant (P = 0.320; S1A Fig). In contrast, there were no differences in the levels of b-FGF in the AMSC group and that of the PBS group (S1B Fig). VEGF was hardly detected from the injured spinal cord in both groups. The AD values were decreased immediately after SCI but showed a better recovery trend in the AMSC group compared with that in the PBS group 3 weeks after SCI (*P* = 0.06; Fig 3E–3G), while RD values did not differ between groups. The results indicate that AMSCs were able to promote neurological recovery by reducing the damaged area and restoring or regaining neuronal fiber networks, particularly in the longitudinal direction in the spinal cord, and neurotrophic factors are partially involved in this recovery.

### AMSCs ameliorated SCI-induced alterations in the intestine

**Fig 4:**
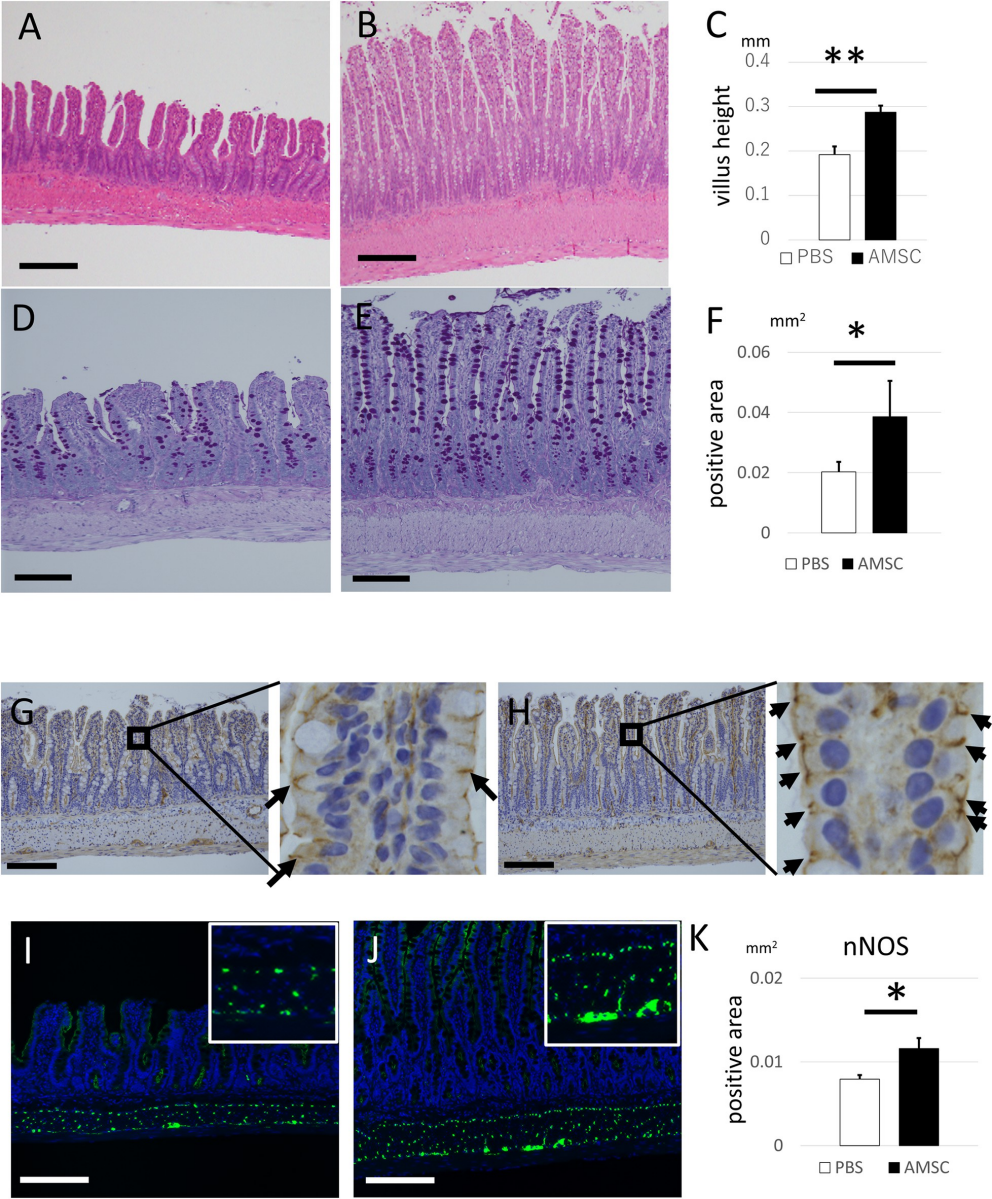
Administration of AMSCs improved intestinal structures, mucus secretion, tight junction, and peristalsis is reversed by spinal-origin neurotransmitter. (A–C) The ileum in PBS group (A) was more atrophic than those in the AMSC group (B) (magnification, ×100; scale bar: 200 μm), and the villus height in the AMSC group was significantly superior to those in the PBS group (C, *P* = 0.0118). (D–F) The ability to produce mucus was evaluated via PAS staining 3 days after SCI. The ileum in PBS group (D) had fewer goblet cells containing intestinal mucus than those in the AMSC group (E) (magnification, ×100; scale bar: 200 μm). The PAS-positive area in the AMSC group was significantly larger than that in the PBS group. (F; *P* = 0.0306). (G–H) The permeability between the intestinal epithelial cells was evaluated via IHC staining using the anti-zo-1 antibody. The zo-1 positive connections between the cells (arrows) in the PBS group (G) were less than those in the AMSC group (H) (magnification, ×100; scale bar: 200 μm). (I–K) Intestinal peristalsis was evaluated 14 d after SCI via the immunofluorescence staining using the anti-nNOS antibody. The nNOS-positive areas in the muscle layers in the PBS group (J) were significantly smaller than those in AMSC group (J) 14 d after SCI (K), (magnification, ×100; scale bar: 200 μm). Data are presented as mean ± standard error (SE). *, *P*<0.05; **, *P*<0.01.

Three days after SCI, the size and density of villi, crypts, and muscle and villus layers of ileum demonstrated severe atrophies in the PBS group; however, AMSC transplantation reversed these atrophies (Fig 4A–4C, S2A–S2C Fig). These recoveries were continuously observed 14 d after SCI in the AMSC group (S2D–S2G Fig). PAS-positive areas were also significantly higher in the AMSC group than in PBS groups 3 and 14 d after SCI (Fig 4D–4F, S2H Fig). The expression of tight junction protein zo-1, was downregulated around the intestinal epithelial cells in the PBS group. In contrast, downregulation was reversed in the AMSC group 3 d post-SCI (Fig 4G and 4H, respectively). There was a higher trend of spinal-cord-oriented neurotransmitter; neuronal nitric oxide synthase (nNOS), in the muscle layer of the ileum in the AMSC group, compared to the PBS group 3 d after SCI (*P* = 0.09, S2I Fig), and it became significantly higher in the AMSC group than in the PBS group 14 d after SCI (Fig 4I–4K). These data showed that transplantation of AMSCs prevented morphological and functional deterioration of the gut in the acute and subacute phases of SCI.

### AMSC administration prevents submucosal bacterial invasion and systemic bacterial translocation

**Fig 5:**
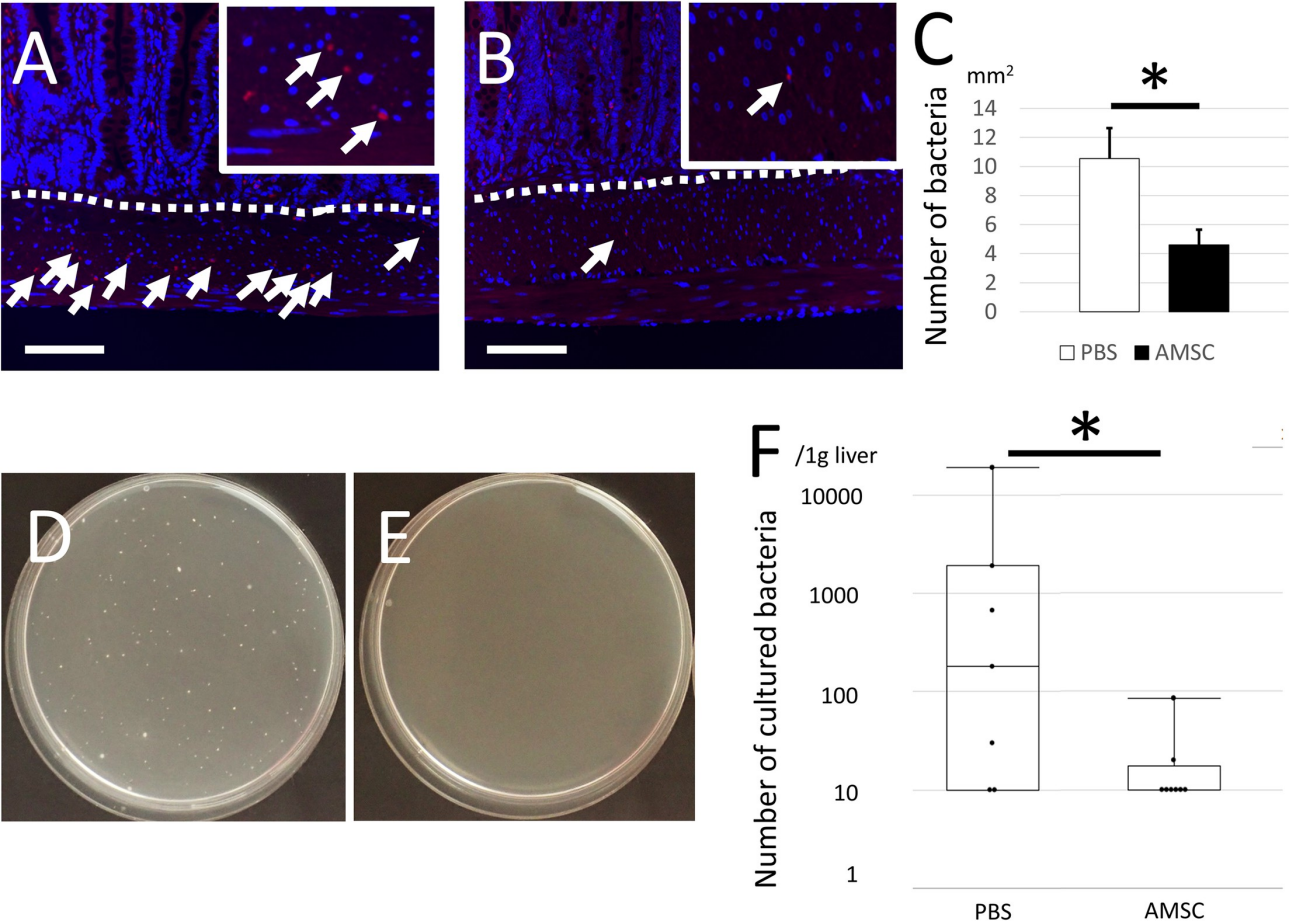
Administration of AMSCs prevented intramucosal and systemic bacterial translocation. (A–C) To evaluate whether the microbiome transferred mucous layer (dotted line) in the intestinal tract, bacterial FISH was performed 3 (S3A–S3C Fig) and 7 d (A–C) after SCI. Although the number of bacterial signals in the lamina propria in PBS groups and AMSC was not significantly different 3 d after SCI, the number of bacterial signals was significantly higher in PBS group (A) than that in AMSC group (B) 7 d after SCI (C), (magnification, ×100; scale bar: 200 μm, *P* = 0.0294). (D–F) The liver bacterial culture 7 d after SCI. The culture dishes in PBS group (D) and AMSC group (E) were shown. The number of bacterial colonies in the AMSC group was significantly smaller than that in the PBS group (F, *P* = 0.0383).

Intestinal bacterial FISH revealed no difference in the numbers of bacterial signals in the lamina propria 3 d after SCI in both groups (S3A–S3C Fig). The signals were increased in PBS group 7 d after SCI, while it was significantly prevented in the AMSC group (Fig 5A–5C). Furthermore, the abundance of bacterial colonies observed in the livers of the PBS group was significantly decreased in the AMSC group 7 d post-SCI (Fig 5D–5F). Although there was a time-dependent change in the intestinal compositions of Bacteroidales and Clostridiales, no significant differences were found between the two groups investigated by 16S rRNA sequencing 3 and 14 d after SCI (S3D and S3E Fig). The Simpson’s indices were similar between groups (S3F and S3G Fig). These data indicate that transplantation of AMSCs prevented the bacterial translocation of gut microbiota from the intestine to the systemic circulation by protecting the functional and morphological function of the gut layer.

### AMSCs do not alter systemic inflammation in the acute phase but suppress spinal and systemic inflammation in the chronic phase

**Fig 6:**
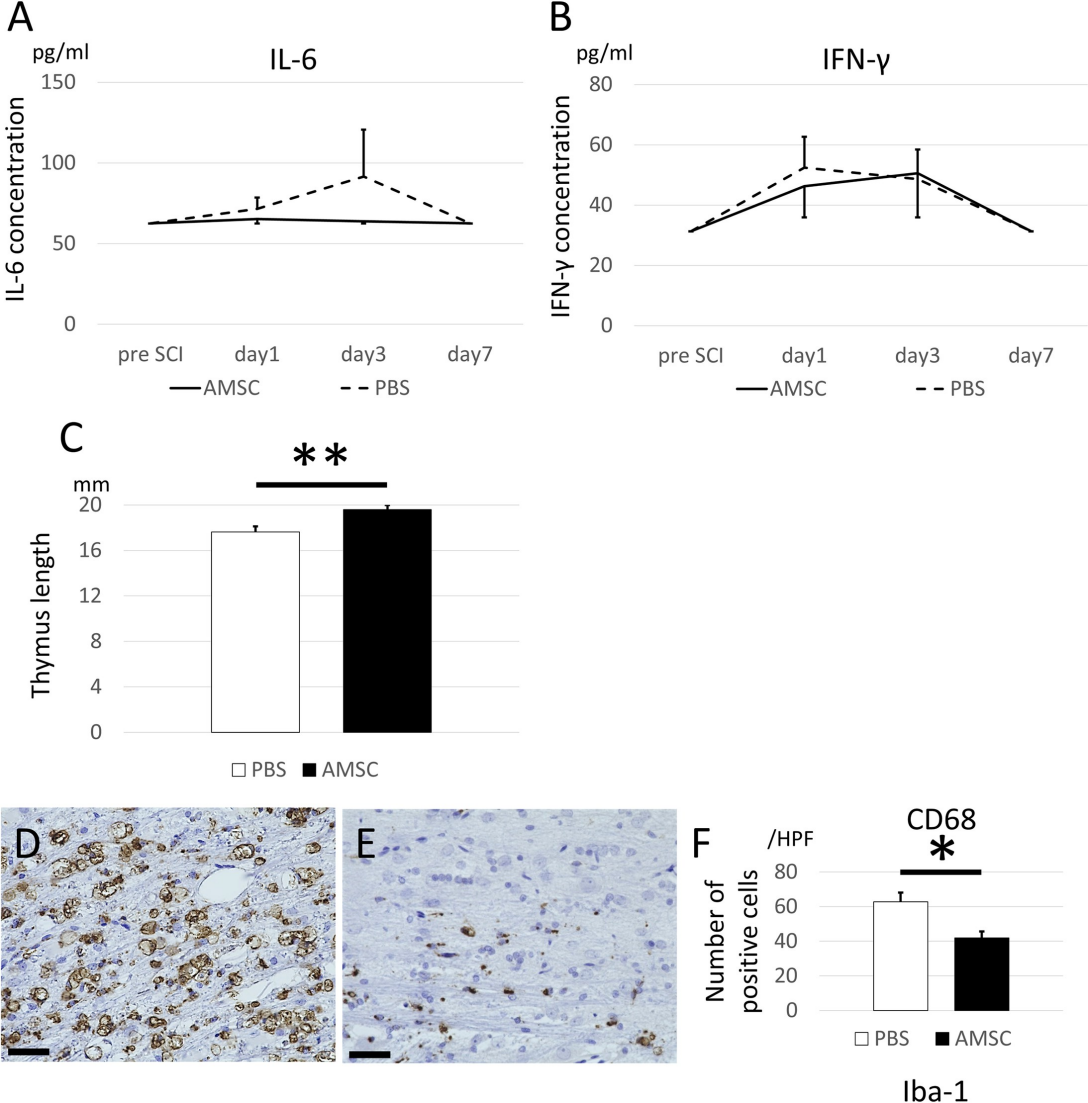
Systemic inflammation in the acute phase was not altered by AMSC transplantation but was reduced in the chronic phase. (A and B) ELISA using blood samples showed that IL-6 (A) and IFN-γ (B) did not increase until 7 d after SCI in both groups. (C) The systemic inflammation in the chronic phase was evaluated by measuring the organ size. The thymus height in the AMSC group was significantly higher than that in the PBS group. (D–F) Inflammation in the spinal cord was assessed via IHC staining using the anti-CD68 antibody. As for CD68, which represents blood-entered macrophage, the number of CD68-positive cells in the PBS group (D) was significantly larger than that in the PBS group (E, F; *P* = 0.0142) (magnification, ×400; scale bar: 20 μm).

IL-6 and IFN-γ expression in the blood revealed that AMSC transplantation did not alter the expression level of acute inflammatory cytokine up to 7 d after SCI (Fig 6A and 6B). However, thymus length in the AMSC group was significantly longer than that of the control group (Fig 6C), and infiltrated macrophages in the spinal cord were significantly reduced in the AMSC group 28 days after transplantation (Fig 6D–6F). Notably, transplantation of AMSCs does not seem to contribute to lowering the systemic inflammation in the acute phase, but does so after the subacute phase.

## Discussion

In the present study, we identified that intravenous transplantation of AMSCs during the acute phase of SCI improved neurological dysfunction. AMSC ameliorated intestinal dysfunction, prevented bacterial translocation, and reduced systemic inflammation in the chronic phase. The prevention of chronic inflammation may partially contribute to late phase neurological recovery.

Stem cell therapy against neurological disorders reportedly acts via two mechanisms, trans-differentiation and the bystander effect [12, 29]. Trans-differentiation refers to the idea that transplanted cells are converted to neuronal or vascular cells to compensate for the loss of the neurological network. The bystander effect pertains to the idea that trophic factors and cytokines released from the cells rescue damaged spinal cord cells, decreasing systemic inflammation. The bystander effect is deemed responsible for the recovery in our case because there were no AMSCs in the damaged spinal cord. These findings were consistent with previous studies on intravenous transplanted MSC cells in SCI models, in which MSCs were seldom found in the damaged area, yet they facilitated neurological recovery [30, 31]. BDNF, VEGF, and b-FGF released from AMSCs ameliorated spinal cord damage. However, HGF and b-NGF, other trophic factors reported to be responsible for neural recovery in spinal cord injury, were not detected [32]. These differences are possibly related to the different cell sources. In the current study, the level of BDNF in the spinal cord was slightly higher in the AMSC group. The increment of BDNF might be derived from the AMSC secretion (human BDNF) and saved spinal cord (rat BDNF). However, it was difficult to distinguish between them because of the homology between human and rat BDNF. Therefore, understanding the precise molecular mechanisms of recovery from trophic factors in the AMSC requires further study.

We found that morphological and functional recovery of the intestines commenced as early as 3 d after SCI, before spinal motor recovery which began two weeks after the injury. Intestinal peristalsis is regulated through the sympathetic nerve, the parasympathetic nerve, and the intrinsic enteric nerve [33], and damage in the spinal cord can cause insufficient communication of sympathetic nerves. Ueno et al. recently showed that inappropriate upregulation of autonomic reflexes in the spinal cord lower than the damaged area provokes an excessive response of sympathetic nerves [7]. It can cause excessive intestinal hypertonic status, eventually impairing the intestinal barrier function. Although the mechanisms by which AMSCs facilitate intestinal recovery need further discussion, AMSCs likely had no direct effect on the intestines according to data of R-Spondin-1 and intestinal cell distribution [13]. One possible mechanism involves the positive effect of AMSCs on the spinal cord, which enables the normalization of the sympathetic nervous system and the improvement of intestinal peristalsis. We evaluated gut neurotransmitters and found that AMSCs increased nNOS expression, a neurotransmitter mainly distributed in the myenteric plexus [34].

We showed that administration of AMSCs resulted in preventing intestinal barrier disruption. Previous reports showed that barrier disruption and dysbiosis impair SCI recovery [9, 10, 25]. Kigerl et al. reported that bacterial translocation occurs approximately seven days from SCI [9]. These data were in agreement with our findings that bacterial translocation was not detectable three days after injury, but was detectable after seven days. Recently, bacterial translocation in the damaged intestine has been reported in the graft-versus-host disease model, which strengthens intestinal permeability to reduce bacterial translocation and systemic inflammatory cytokines and ameliorates graft-versus-host disease. We found that bacterial culture was prevented in the AMSC transplanted group, which might have contributed to the inhibition of additional inflammatory action seen in the thymus atrophy. Another explanation of systemic inflammation caused by bacterial translocation is chemokines produced by bacteria or bacterial components. Bacterial translocation and production of local proinflammatory chemokines are closely related [35], and CC chemokine ligand-3, CC chemokine receptor-1, and CC chemokine receptor-5 were increased after SCI [36]. Bacterial translocation and a local chemokine increase are reported to increase macrophage activation [37], leading to the aggravation of local inflammation.

There are several limitations to this study. First, the precise mechanisms underlying how stem cell therapy positively affected intestinal function remain unclear. We found that nNOS expression was upregulated. However, the underlying mechanism of the spinal cord behind this effect remains elusive. Cutting all efferent nerves originating from the spinal cord to the intestine can directly prove the relationship of the spinal-gut axis. However, this was technically impossible in small rodents, whose nerves and ganglia are difficult to find with subtle surgical procedures that might cause further intestinal damage such as adhesion. Histopathological examination of cholinergic receptors in the spinal cord may also prove the relationship between the spinal cord and intestinal function. Second, we did not prove that bacterial translocation directly exacerbated chronic inflammation and aggravated spinal cord damage. Strengthening the intestinal barrier function or eliminating the whole gut microbiome could help address this issue. Evaluation of inflammatory cytokines, as well as regulatory T cells at the chronic phase, is important in elucidating the functional mechanisms involved in this process.

## Conclusion

Mesenchymal stem cells (MSC) reportedly ameliorate SCI. In this study, we investigated their effect on the associated gastrointestinal dysfunction. Our findings provide strong evidence of the usefulness of AMSC administration during the acute phase of SCI. AMSC administration preserves intestinal barrier function, prevents bacterial translocation, and ameliorates neurological damage. Since AMSC can be mass-produced from an allogenic source, our findings can potentially be a breakthrough in SCI treatment.

## Supporting information

S1 Fig Neurotrophic factors in the spinal cord after AMSCs transplantation. The level of BDNF in spinal cord in AMSC group was slightly higher than that in the PBS group, although it was not statistically significant (P = 0.320, A). In contrast, there were no differences for the levels of b-FGF between the AMSC and PBS group (B).
(TIF)

S2 Fig Administration of AMSCs improved intestinal structures, mucus secretion, tight junction, and peristalsis is reversed by spinal-origin neurotransmitter. Intestinal structural changes were evaluated via H&E staining 3 d (A–C) and 14 d (D–G) after SCI. After 3 d, crypt depth (A) and muscle thickness (B) in the AMSC group were significantly superior to those in the PBS group (*P* = 0.0055, 0.0157, respectively), while the villus density (C) was not significantly different between the two groups at this point. After 14 d, the ileum in the PBS group was sustainably more atrophic than those in the AMSC group, that the villus height (D), crypt depth (E), muscle thickness (F), and the villus density (G) in AMSC group were significantly higher than those in PBS group (*P* = 0.0118, 0.0081, 0.0055, 0.0115, respectively). The ability to produce mucus was evaluated via PAS staining 14 d (H) after SCI. The ileum in the PBS group still had fewer goblet cells than those in the AMSC group, and the PAS-positive area in the AMSC group was significantly larger than that in the PBS group (*P* = 0.0169). Intestinal peristalsis was evaluated 3 d after SCI via the immunofluorescence staining. n-NOS-positive areas in the muscle layers in the PBS group and AMSC group were not significantly different 3 d after SCI (I) Data are presented as mean ± standard error (SE). *, *P*<0.05; **, *P*<0.01.
(TIF)

S3 Fig Invasion of microbiome to the intestinal mucosa in the acute phase, and the change of bacterial flora in the intestine (A–C) To evaluate whether the microbiome transferred mucous layer (dotted line) in the intestinal tract, bacterial FISH was performed 3 d after SCI. The number of bacterial signals in the lamina propria in PBS groups (A) and AMSC (B) were not significantly different 3 d after SCI (C) (magnification, ×100; scale bar: 200 μm). (D–G) The percentages of Bacteroidales (J) and Clostridiales (K) were not significantly different 3 and 14 d after SCI. Moreover, the microbial diversities based on the Simpson’s index between the AMSC and PBS groups were not significantly different 3 (F) and 14 d (G) after SCI.
(TIF)

S1 File (DOCX)
