# Supplementary figures and images for: Intravenous transplantation of amnion-derived mesenchymal stem cells promotes functional recovery and alleviates intestinal dysfunction after spinal cord injury
Source: PLoS One. 2022 Jul 8;17(7):e0270606. doi: 10.1371/journal.pone.0270606 (PMC9269969; doi:10.1371/journal.pone.0270606)

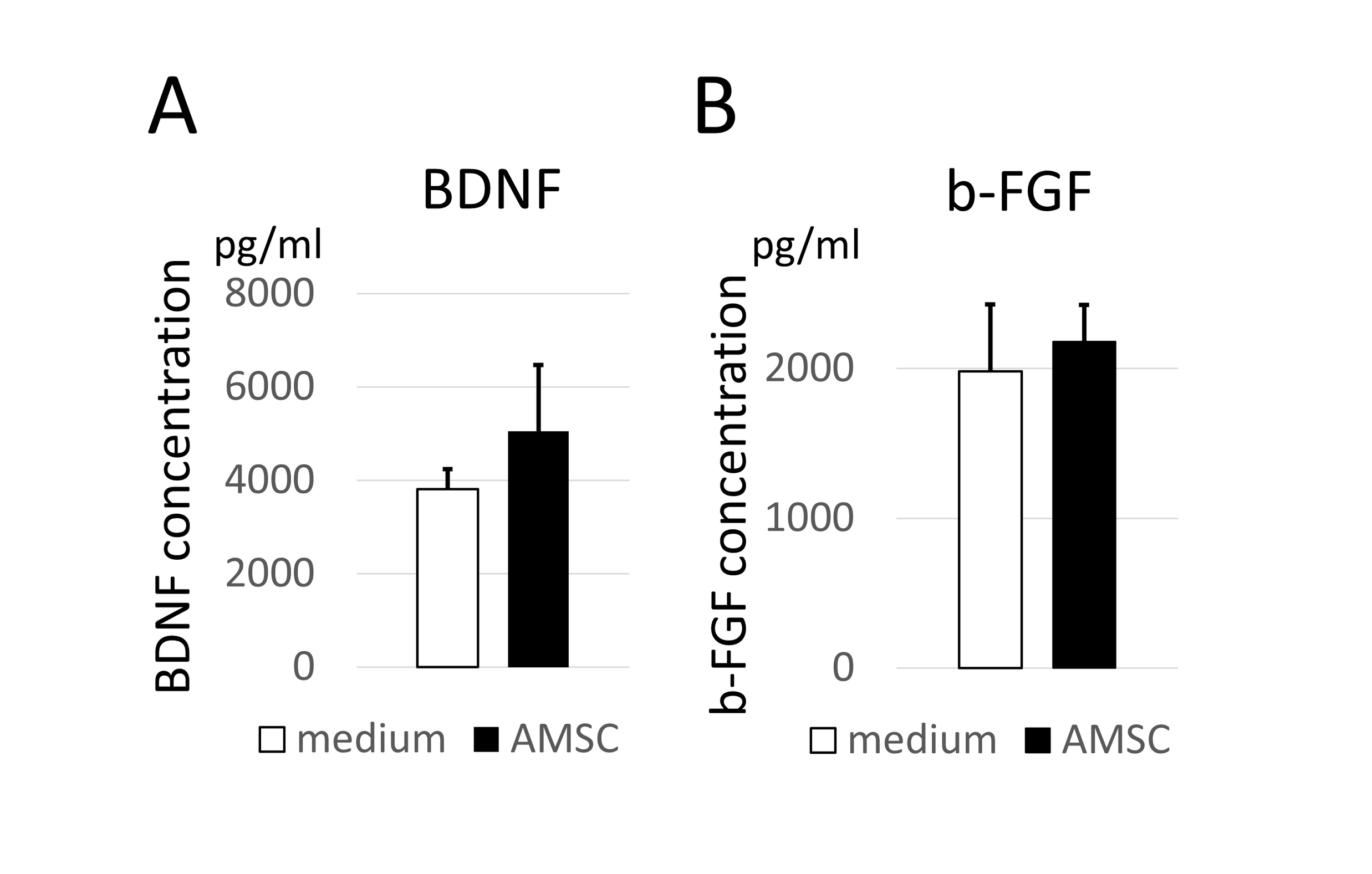

Supplement: S1 Fig — The level of BDNF in spinal cord in AMSC group was slightly higher than that in the PBS group, although it was not statistically significant (P = 0.320, A). In contrast, there were no differences for the levels of b-FGF between the AMSC and PBS group (B). (TIF) [file pone.0270606.s001.tif]

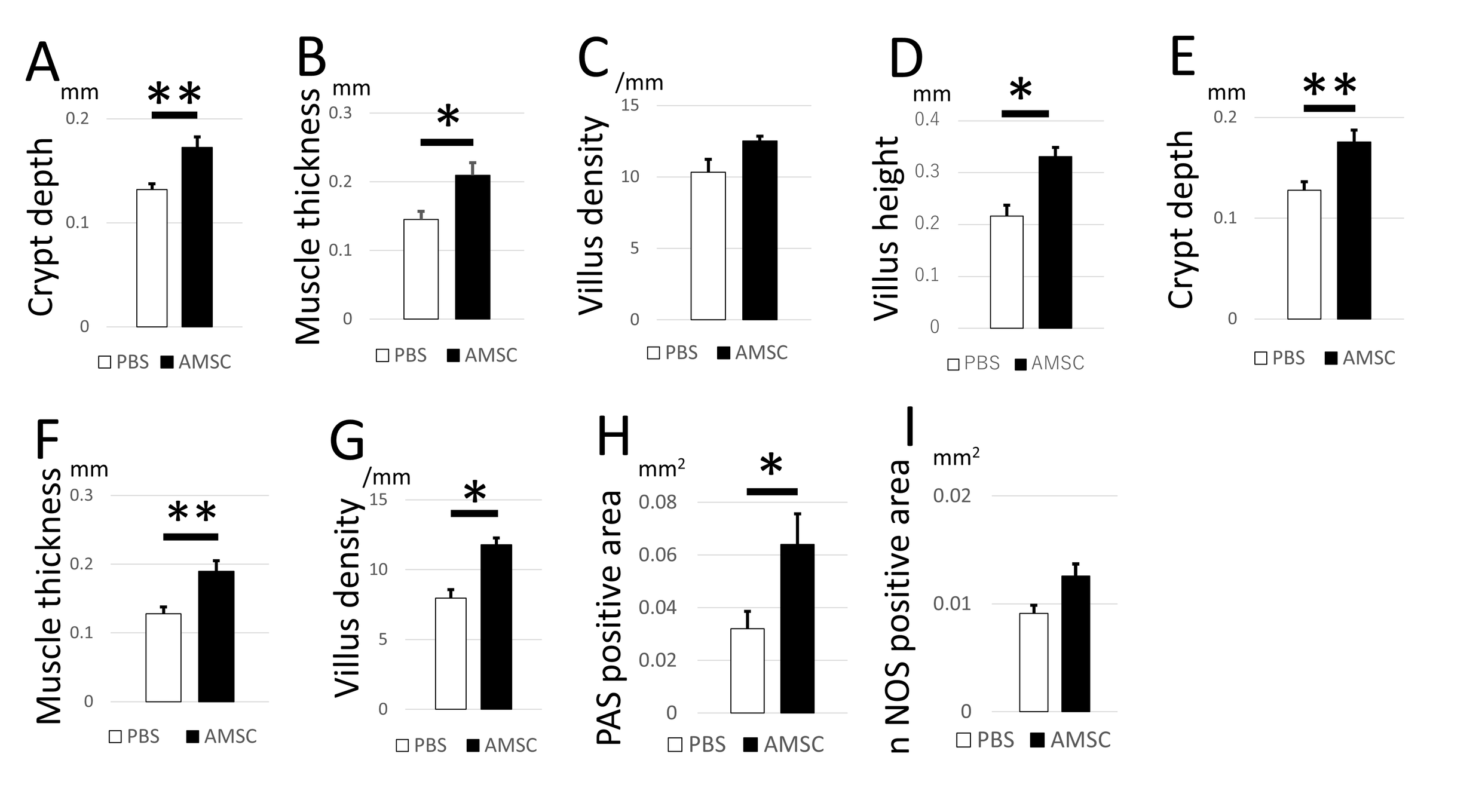

Supplement: S2 Fig — Intestinal structural changes were evaluated via H&E staining 3 d (A–C) and 14 d (D–G) after SCI. After 3 d, crypt depth (A) and muscle thickness (B) in the AMSC group were significantly superior to those in the PBS group (P = 0.0055, 0.0157, respectively), while the villus density (C) was not significantly different between the two groups at this point. After 14 d, the ileum in the PBS group was sustainably more atrophic than those in the AMSC group, that the villus height (D), crypt depth (E), muscle thickness (F), and the villus density (G) in AMSC group were significantly higher than those in PBS group (P = 0.0118, 0.0081, 0.0055, 0.0115, respectively). The ability to produce mucus was evaluated via PAS staining 14 d (H) after SCI. The ileum in the PBS group still had fewer goblet cells than those in the AMSC group, and the PAS-positive area in the AMSC group was significantly larger than that in the PBS group (P = 0.0169). Intestinal peristalsis was evaluated 3 d after SCI via the immunofluorescence staining. n-NOS-positive areas in the muscle layers in the PBS group and AMSC group were not significantly different 3 d after SCI (I) Data are presented as mean ± standard error (SE). *, P<0.05; **, P<0.01. (TIF) [file pone.0270606.s002.tif]

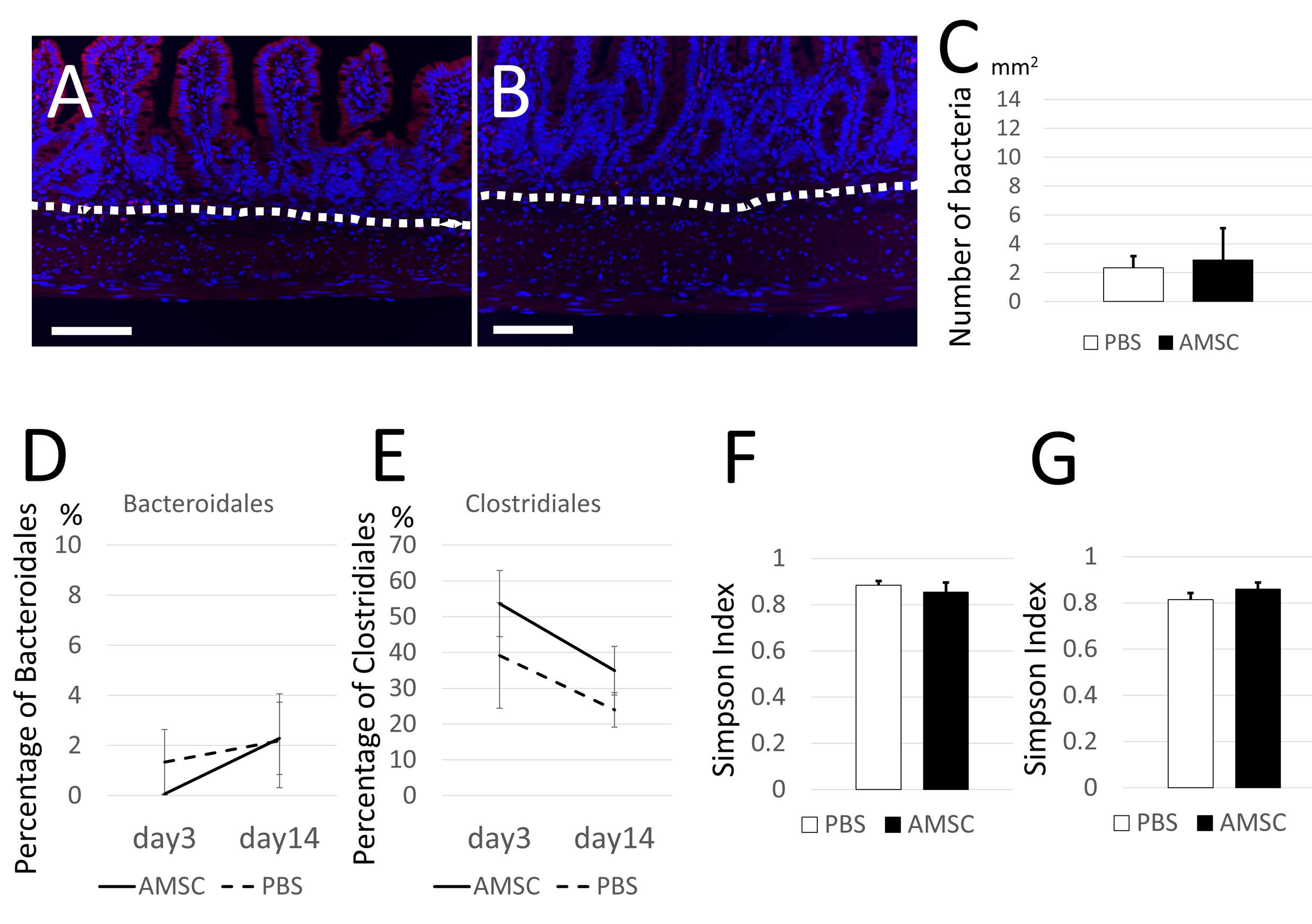

Supplement: S3 Fig — The number of bacterial signals in the lamina propria in PBS groups (A) and AMSC (B) were not significantly different 3 d after SCI (C) (magnification, ×100; scale bar: 200 μm). (D–G) The percentages of Bacteroidales (J) and Clostridiales (K) were not significantly different 3 and 14 d after SCI. Moreover, the microbial diversities based on the Simpson’s index between the AMSC and PBS groups were not significantly different 3 (F) and 14 d (G) after SCI. (TIF) [file pone.0270606.s003.tif]
