# Supplementary material for: Intravenous transplantation of amnion-derived mesenchymal stem cells promotes functional recovery and alleviates intestinal dysfunction after spinal cord injury
Source: PLoS One. 2022 Jul 8;17(7):e0270606. doi: 10.1371/journal.pone.0270606 (PMC9269969; doi:10.1371/journal.pone.0270606)
Supplement: S1 File — (DOCX) [file pone.0270606.s004.docx]

**Supplemental Data**

**Material and Methods**

**AMSC preparation and measurement of trophic factors**

AMSCs were generated from amniotic membrane obtained from healthy pregnant volunteers with written informed consent. The membrane was digested with collagenase and dispase and cultured in an uncoated culture chamber with MEM α (Thermo Fisher Scientific, MA, USA) supplemented with 1% antibiotic-antimycotic (Thermo Fisher Scientific) and 5% human platelet lysate. They were then incubated at 37 ℃ in a humidified atmosphere containing 5% CO_2_ [1, 2]. After reaching confluence, cells were detached with trypsin and cryopreserved at -80 ℃ in liquid nitrogen until use. Five days before transplantation, the frozen cryovials were thawed. Cells were re-cultured without passages under the aforementioned conditions and detached with trypsin immediately before transplantation.

For ELISA evaluation, AMSCs were quantified using commercially available ELISA kits (total BDNF, human HGF, human VEGF, human bFGF, and human R-Spondin 1; R&D Systems, Inc., MN, US; human β-NGF; RayBiotech, Inc., GA, US) according to the manufacturer’s protocols.

**Animals, SCI model, and cell transplantation**

A total of 114 animals were examined. The rats were housed in a controlled environment (23±3 ℃, 50±10% humidity) under a 12-h light/12-h dark cycle with free access to food and water. For the SCI model, after the induction of general anesthesia with 5% isoflurane in 70% N_2_O and 30% O_2_ gas, followed by the maintenance of anesthesia with 1.5–2% isoflurane in 70% N_2_O and 30% O_2_ gas, rats were placed in the prone position. After midline skin incision, T6–7 vertebral arches were exposed, and T6–7 laminectomy was performed. The spinal cord was extradurally pinched at the T6/7 level via the modified aneurysm clip for 1 min, and the skin was sutured. After SCI, bladders were gently pressed three times daily for the first 2 weeks and two times daily after the third week to force urination. After 24 h, the motor function of the hind limbs was evaluated using the Basso-Beattie-Bresnahan (BBB) scale, and six animals that showed BBB scores of more than 2 were excluded from this study because they represented incomplete damage.

For in vivo imaging for the cells’ distribution, six rats were fed with Alfalfa-free foods (iVid-neo, Oriental Yeast Co. Ltd., Japan) for 2 weeks before SCI to reduce autofluorescence. A day after SCI, AMSCs were co-incubated with 31.6 mM of XenoLight DiR (Perkin Elmer, Inc., MA, USA) for 30 min at 37 ℃ and washed twice with PBS. Three rats were transplanted with 1 × 10^7^ AMSCs, whereas the others were injected with PBS. Rats were anesthetized with 1.5–2% isoflurane in 70% N_2_O and 30% O_2_ gas and placed on supine and prone positions in the imaging device. The excitation and emission filters were 680 nm and 750 nm, respectively. The exposure times were unified into 0.5 s. One rat in the AMSC group was euthanized after the first imaging to conduct *ex vivo* imaging, while the others were euthanized 7 d after transplantation.

Immunohistochemistry (IHC) staining was also performed using the anti-Ku80 antibody. Approximately 5-μm-thick sections were incubated with the anti-Ku80 antibody for 1 h at room temperature. After the first antibody incubation, sections were treated with Histofine Simple Stain MAX-PO (Nichirei Biosciences Inc., Tokyo, Japan) for 30 min and reacted with the 3,3’-diaminobenzidine (DAB) (Simple Stain DAB Solution, Nichirei Biosciences Inc.) for 3 min.

**Neurological, histological, and radiographical evaluation of spinal cord after cell transplantation**

The BBB score mentioned above, which ranged from 0 (complete paraplegia) to 21 (normal hindlimbs movement), was evaluated for 5 min in the open field. The individual score represented the average of bilateral scores performed by investigators (S. T. and K. Y.) blinded to the experimental groups.

The spinal cord was collected for pathological assessment as previously described[3]. They were collected 28 d after SCI. After anesthetizing the rats, they were transcardially perfused with cold saline and by 4% paraformaldehyde (PFA) consequently. The spinal cord, including the injured lesion, was fixed with 4% PFA for 24 h. They were then embedded in paraffin and cut using a manual microtome (LEICA RM2125 RTS, Leica Biosystems, Germany). Section thicknesses were 10 µm for the Kluver-Barrera staining[3]. For Kluver-Barrera staining, sagittal spinal cord sections were incubated in Luxol fast blue (LFB) stain solution (Muto Pure Chemicals Co. Ltd., Tokyo, Japan) at 60 ℃ for 15 h. They were decolorized with 0.1% lithium carbonate solution (Muto Pure Chemicals Co. Ltd.) for 10 s and with 70% ethanol for 3 min. The sections were then incubated in 0.1% cresyl violet solution (Muto Pure Chemicals Co. Ltd.) for 5 min.

The length of the injured lesion was measured by referring to the absence of the LFB stain.

The spinal cords were collected, for trophic factor assessment *in vivo*, 3 d after SCI (n=6). After the induction of general anesthesia, rats were transcardially perfused with cold saline and the thoracic vertebrae, including the injured spinal cord were removed. The spinal cords were carefully collected, cut into 10-mm length sections with a central focus on the lesions, and immediately stored at −80℃. The samples were homogenized in RIPA lysis buffer with protease inhibitor (Santa Cruz Biotechnology, Inc., TX, USA) using the bead mill benchtop homogenizer (Shakeman 3; BioMedical Science Co., Ltd, Tokyo, Japan). The homogenates were centrifuged at 10,000 ×g for 10 min, and the supernatants were collected for analysis. The concentrations of trophic factors were quantified using commercially available ELISA kits (total BDNF, human VEGF, and human bFGF; R&D Systems, Inc., MN, USA) according to the manufacturer’s protocols.

For radiological evaluation, rats were anesthetized and placed into an eight-channel birdcage coil (Takashima Seisakusho Co. Ltd., Japan) for rodent models. Diffusion tensor imaging (DTI) was performed using a single-shot spin-echo echo-planar sequence under the following scan parameters: repetition time/ echo time (TR/TE) = 5,000/ 49 ms; voxel size = 0.5 × 0.5 × 0.0632 mm^3^; the number of excitations = 1; b-value = 0 and 700 s/mm^2^; the number of gradient directions = 60; and plane = axial. Sagittal T2-weighted images (TR/TE = 6,000/96 ms; voxel size = 0.5 × 0.5 × 0.0632 mm^3^) were also obtained for anatomical information including confirmation of vertebral level. From the DTI data, fractional anisotropy (FA), axial diffusivity (AD), and radial diffusivity (RD) maps were generated (Dr. View/ INUX R2.5.0; AJS, Tokyo, Japan). FA refers to the anisotropy of diffusion, AD represents the directional diffusivity along the axonal pathway, and RD represents the diffusivity along the orthogonalized axonal pathway [4]. These maps were then co-registered to the sagittal T2-weighted images to identify the vertebral level (SPM12, Wellcome Trust Centre for Neuroimaging, University College of London, Oxford, UK). Next, a free-hand region of interest (ROI) was drawn around the spinal cord at the level of T4 through T9 on the co-registered T2-weighted images (MRICron Version 1, www.mricro.com). The ROI was then applied to the co-registered DTI maps as an inclusion mask. The threshold of the inclusion mask was set to within five standard deviations below the mean FA at the pre-SCI state to prevent the inclusion of voxels other than the spinal cord (ImageJ 1.52a, National Institutes of Health, Bethesda, MD, USA). For each ROI, the major FA, AD, and RD histogram metrics (mean, minimum, maximum, and mode) were extracted (Image J 1.52a).

**Histological analysis of gut after SCI with/without cell transplantation**

The ileum was collected 3 d (n = 24), 7 d (n = 8), and 14 d (n = 14) after SCI for pathological assessment. After anesthetizing the animals, they were transcardially perfused with cold saline and by 4% PFA consequently. Approximately 5 cm of the ileum were collected 5 cm above the ileocecal valve and fixed with 4% PFA for 24 h. They were then embedded in paraffin and cut using a manual microtome (Leica Biosystems, Germany). Section thicknesses were 5-µm.

For H&E staining, the longitudinal ileum sections were incubated in hematoxylin (Muto Pure Chemicals Co. Ltd.) for 5 min and in eosin (Muto Pure Chemicals Co. Ltd.) for 4 min. For each slide, the villus height, villus density (the number of villi per millimeter), crypt depth, and muscle layer thickness were measured using 10 well-oriented villi/crypt units[5].

For PAS staining, the longitudinal ileum sections were incubated in 0.5% PAS for 10 min. Thereafter, the sections were subjected to the reaction with a self-prepared Schiff’s reagent for 15 min and incubated in hematoxylin solution (Muto Pure Chemicals Co. Ltd.) for 30 s. For each slide, PAS-positive areas were evaluated using an automated area counter (BZ-X Analyzer, Keyence Co., Osaka, Japan) under a magnification of 100× in the five non-overlapped fields[5].

IHC staining using anti-zo-1 antibody was performed with the same protocol mentioned above[6].

Immunofluorescence staining was also performed using anti-nNOS antibody[7]. Approximately 5-μm-thick longitudinal ileum sections were incubated with anti-nNOS antibody overnight at 4 ℃. The sections were then treated with Histofine Simple Stain MAX-PO (Nichirei Biosciences Inc., Tokyo, Japan) for 30 min. They were then subjected to the TSA Fluorescein System (SAT701001EA; Akoya Biosciences, MA, USA) for 10 min to 4’,6-diamido-2-phenylindole (DAPI, D1306; Invitrogen Life Technologies) for 1 min.

**Bacterial translocation analysis and evaluating systemic inflammation**

Fluorescence in situ hybridization (FISH) was performed as previously described [8]. 5-μm-thick longitudinal ileum sections were incubated with 1 µg Cy5-conjugated universal bacterial probe, EUB338 (5′-GCTGCCTCCCGTAGGAGT-3′), overnight at 40 ℃. The sections were then washed at 45 ℃ for 20 min in wash buffer (50 mM NaCl, 4 mM Tris-HCl, 0.05 mM EDTA) and enclosed using a mounting agent containing DAPI. Each slide was observed under a magnification of 200×. The exposure time was 0.2 s. For each slide, the number of bacterial signals in the lamina propria, which was considered to invade through the inner mucus layer, was counted in the five non-overlapped fields.

For the bacterial culture, the liver was collected with an aseptic procedure 7 d after SCI. The collected organs were determined based on previous research [9] and our preliminary experiments. The collected sample was homogenized with sterilized saline and diluted 10 times with sterilized saline. The serial dilution was performed in the same procedure. Approximately 1 mL of each diluted sample was poured into the sterile disposable dish with 15 mL of sterilized Standard Method Agar (Nissui, Tokyo, Japan). They were then incubated for 48 h at 37 ℃ in aerobic conditions. After the incubation, the number of colonies was manually counted.

To assess the gut microbiota after SCI, small intestinal contents were collected into sterile tubes at the same time that the intestinal tract was harvested. The samples were frozen at -80 ℃. After freeze-drying, the samples were soaked into Lysis Solution F (Nippon Gene Co., Ltd., Tokyo, Japan) for 10 min at 65 ℃ and centrifuged thereafter. Total DNA was extracted from the supernatants using the MPure Bacterial DNA Extraction Kit (MP Biochemicals, CA, USA). Two-step tailed PCR was performed to amplify the V1/V2 hypervariable regions (the forward and reverse primers were set on 27 and 338) of the 16S rRNA gene using the MiSeq Reagent Kit V3 (Illumina, CA, USA) following the 2 × 300 base pair ended protocol. The sequencing data were extracted for analysis using the fastq_barcode_spliltter of Fastx toolkit (ver. 0.0.14, <http://hannonlab.edu/fastx-toolkit>). The primers, chimera, and noise sequences were filtered out using the Qiime 2 (ver. 2020.8, <https://qiime2.org>). The representative sequences were then identified. The obtained sequences were classified into species-equivalent operational taxonomic units based on more than 97% similarity by comparing with operational taxonomic units in the Greengenes database. The microbial diversity was analyzed using the Qiime and the Simpson’s index of each sample, which were calculated.

To evaluate systemic inflammation in the acute phase, the inflammation cytokines were measured over time until 7 d after SCI using blood samples collected from the tail veins (n = 4–8, AMSC group and PBS group, respectively). IL-6 and IFN-γ were quantified using ELISA kits (rat IL-6 and rat IFN-γt; R&D Systems, Inc., MN, US) according to the manufacturer’s protocols.

1. Kobayashi K, Ichihara Y, Sato N, Umeda N, Fields L, Fukumitsu M, et al. On-site fabrication of Bi-layered adhesive mesenchymal stromal cell-dressings for the treatment of heart failure. Biomaterials. 2019;209:41-53. Epub 2019/04/27. doi: 10.1016/j.biomaterials.2019.04.014. PubMed PMID: 31026610; PubMed Central PMCID: PMCPMC6527869.

2. Tago Y, Kobayashi C, Ogura M, Wada J, Yamaguchi S, Yamaguchi T, et al. Human amnion-derived mesenchymal stem cells attenuate xenogeneic graft-versus-host disease by preventing T cell activation and proliferation. Sci Rep. 2021;11(1):2406. Epub 2021/01/30. doi: 10.1038/s41598-021-81916-y. PubMed PMID: 33510297; PubMed Central PMCID: PMCPMC7843654.

3. Yamazaki K, Kawabori M, Seki T, Takamiya S, Tateno T, Konno K, et al. FTY720 Attenuates Neuropathic Pain after Spinal Cord Injury by Decreasing Systemic and Local Inflammation in a Rat Spinal Cord Compression Model. J Neurotrauma. 2020;37(15):1720-8. Epub 2020/03/29. doi: 10.1089/neu.2019.6905. PubMed PMID: 32216535; PubMed Central PMCID: PMCPMC7368387.

4. Zhao C, Rao JS, Pei XJ, Lei JF, Wang ZJ, Zhao W, et al. Diffusion tensor imaging of spinal cord parenchyma lesion in rat with chronic spinal cord injury. Magn Reson Imaging. 2018;47:25-32. Epub 2017/11/21. doi: 10.1016/j.mri.2017.11.009. PubMed PMID: 29154896.

5. Onishi S, Kaji T, Yamada W, Nakame K, Machigashira S, Kawano M, et al. Ghrelin stimulates intestinal adaptation following massive small bowel resection in parenterally fed rats. Peptides. 2018;106:59-67. doi: 10.1016/j.peptides.2018.06.009. PubMed PMID: 29966680.

6. Wang Z, Higashikawa K, Yasui H, Kuge Y, Ohno Y, Kihara A, et al. FTY720 Protects Against Ischemia-Reperfusion Injury by Preventing the Redistribution of Tight Junction Proteins and Decreases Inflammation in the Subacute Phase in an Experimental Stroke Model. Transl Stroke Res. 2020;11(5):1103-16. doi: 10.1007/s12975-020-00789-x. PubMed PMID: 32103462; PubMed Central PMCID: PMCPMC7496052.

7. Kabatas S, Yu D, He XD, Thatte HS, Benedict D, Hepgul KT, et al. Neural and anatomical abnormalities of the gastrointestinal system resulting from contusion spinal cord injury. Neuroscience. 2008;154(4):1627-38. Epub 2008/06/17. doi: 10.1016/j.neuroscience.2008.04.071. PubMed PMID: 18556138.

8. Ara T, Hashimoto D, Hayase E, Noizat C, Kikuchi R, Hasegawa Y, et al. Intestinal goblet cells protect against GVHD after allogeneic stem cell transplantation via Lypd8. Sci Transl Med. 2020;12(550). doi: 10.1126/scitranslmed.aaw0720. PubMed PMID: 32611682.

9. Kigerl KA, Hall JC, Wang L, Mo X, Yu Z, Popovich PG. Gut dysbiosis impairs recovery after spinal cord injury. J Exp Med. 2016;213(12):2603-20. doi: 10.1084/jem.20151345. PubMed PMID: 27810921; PubMed Central PMCID: PMCPMC5110012.
